# Minimization of polymerization shrinkage effects on composite resins by the control of irradiance during the photoactivation process

**DOI:** 10.1590/1678-7757-2017-0528

**Published:** 2018-05-29

**Authors:** Gabriel Felipe GUIMARÃES, Edilmar MARCELINO, Ivana CESARINO, Fábio Bossoi VICENTE, Carlos Roberto GRANDINI, Rafael Plana SIMÕES

**Affiliations:** 1 Univ. Estadual Paulista, Faculdade de Ciências Agronômicas, Departamento de Bioprocessos e Biotecnologia, Botucatu, SP, Brasil.; 2 Univ. Estadual Paulista, Instituto de Biociências, Botucatu, SP, Brasil.; 3 Univ. Estadual Paulista, Faculdade de Ciências, Laboratório de Relaxações Anelásticas e Biomateriais, Bauru, SP, Brasil.; 4 Instituto de Biomateriais, Tribocorrosão e Nanomedicina - Ramo Brasileiro, Bauru, SP, Brasil.

**Keywords:** Dental restoration, Composite resins, Light-curing of dental adhesives

## Abstract

High levels of shrinkage stress caused by volumetric variations during the activation process are one of the main problems in the practical application of composite resins.

**Objective:**

The aim of this study is to reduce the shrinkage stress and minimize the effects caused by composite resin volumetric variation due to the photopolymerization. In this way, this work proposes a systematic study to determine the optimal dimming function to be applied to light curing processes.

**Material and Methods:**

The study was performed by applying mathematical techniques to the optimization of nonlinear objective functions. The effectiveness of the dimming function was evaluated by monitoring the polymerization shrinkage stress during the curing process of five brands/models of composites. This monitoring was performed on a universal testing machine using two steel bases coupled in the arms of the machine where the resin was inserted and polymerized. The quality of the composites cured by the proposed method was analyzed and compared with the conventional photoactivation method by experiments to determine their degree of conversion (DC). Absorbance measurements were performed using Fourier-transform infrared spectroscopy (FT-IR). A T-test was performed on DC results to compare the photoactivation techniques. We also used scanning electron microscopy (SEM) to analyze *in-vitro* the adhesion interface of the resin in human teeth.

**Results:**

Our results showed that the use of the optimal dimming function, named as exponential, resulted in the significant reduction of the shrinkage stress (~36.88% ±6.56 when compared with the conventional method) without affecting the DC (t=0.86, p-value=0.44). The SEM analyses show that the proposed process can minimize or even eliminate adhesion failures between the tooth and the resin in dental restorations.

**Conclusion:**

The results from this study can promote the improvement of the composite resin light curing process by the minimization of polymerization shrinkage effects, given an operational standardization of the photoactivation process.

## INTRODUCTION

The composite resins emergence has provided higher aesthetic quality in dental restorations. However, the mechanical properties of the resins, especially the high levels of shrinkage stress caused by volumetric variations during the activation process, are the main problem in the practical application of these materials^2^ ^,^ ^5^.

Despite the chemical advances in resins for the minimization of the polymerization shrinkage, this problem persists and can allow micro-infiltrations in the adhesive interface, which harm the restoration aesthetics and, in some extreme cases, favor the growth of bacteria due to marginal leakage^4^.

To achieve both efficiency and quality, various efforts have been made to reduce the effects of polymerization shrinkage. Current approaches include the improvement of the chemical composition of the resins, the photo-initiator molecules and the polymerization process catalysts^3^ ^,^ ^11^ ^,^ ^17^.

However, studies have shown that the variation of some physical parameters can significantly change the resin shrinkage during the curing process, concluding that the radiant exitance (or emittance) of the curing device has a correlation with the volumetric contraction induction of photopolymerized composite resins^9^ ^,^ ^15^ ^,^ ^18^. It has been observed a variation of shrinkage stress when the composite is polymerized under different conditions of exposure to the luminous source, being that some studies showed that the irradiance modulation during the photoactivation process has an influence on the shrinkage stress modulus^10^.

Alternative light-activation protocols have been proposed to minimize the polymerization shrinkage effects^2^ ^,^ ^10^ ^,^ ^17^ ^-^ ^19^. The soft-start techniques, for example, have been extensively studied. In this kind of photoactivation, the polymerization is started with low irradiance for a few seconds, followed by increased irradiance for the remaining period of light-activation. This process advocated as an approach to reduce the shrinkage stress while maintaining proper degree of conversion (DC) of the composite^3^. However, there is no consensus about the time and irradiance parameters in the soft-start techniques. Therefore, in the literature, it is possible to find soft-start protocols with different values of irradiance and activation time, both for the initial phase of low irradiance and for the final phase of high irradiance^16^. In addition, this type of study is limited to the functions of conventional light curing devices, which do not allow continuous variation of the irradiance during the activation.

Our hypothesis is that it can be possible to determine an optimal curve of irradiance variation during the photoactivation, which will promote the reduction of the shrinkage stress without affecting the cured composites DC.

Considering the above, this project proposes a systematic study to optimize the dimming function in light curing processes. The study was performed by a mathematical approach to determine an optimal function for the irradiance modulation at the time domain. This function was incorporated in a light curing unit, developed by our research group, which can control the irradiance according to a mathematical function. The effectiveness of the optimal function was evaluated by monitoring the polymerization shrinkage stress during the curing process. The quality of the resins polymerized by the proposed method was experimentally analyzed to determine the conversion degree of the monomers into polymers. The interface adhesion between the teeth and resins was analyzed by scanning electron microscopy (SEM).

## Materials and methods

This work has three major stages of development: (1) determination of an optimal function for the irradiance modulation using data from the variation pattern of the shrinkage stress in photoactivation conventional processes, aiming to minimize the effects of the polymerization shrinkage; (2) evaluation of the cured composite quality by monitoring and comparing the shrinkage stress during the polymerization and the DC for the conventional and proposed photoactivation methods and; (3) the use of SEM to evaluate the adhesion interface between teeth and resins by also comparing the conventional and proposed methods.

For all experiments, the following composite resins were analyzed: Z250 (3M Dental Products; St. Paul, Minnesota, USA – Lot 386222, color A2); Z350 (3M Dental Products; St. Paul, Minnesota, USA – Lot 545195, color A2D); Charisma (Heraeus Kulzer; Hanau, Hesse, Germany – Lot 010625A, color A3); NT Premium (Coltene; Altstatten, St. Gallen, Switzerland – Lot 1501559, color A2) and Ultrafill (Biodinâmica; Ibiporã, Paraná, Brazil – Lot 43815, color A2).

All the photoactivation processes were performed using a polymerization unit developed by our research group (patent registration number BR1020160078245, INPI, Brazil). This device was equipped with a second generation Light Emitting Diode (LED) (LZ4-40B208-0000, LED Engin Inc; San Jose, California, USA). The LED device has a typical relative spectral power distribution in wavelength range of 430–480 nm (with peak at 457 nm) and power rating of 10 W. This device was coupled with an optical tip (diameter of 8 mm) and the maximum output irradiance was adjusted to be 1000 mW/cm^2^, which was calibrated using a radiometer (RD7, Ecel Indústria e Comércio Ltda; Ribeirão Preto, São Paulo, Brazil). The developed device was connected to a personal computer containing a computational interface that allows the control of the LED emittance along the photoactivation process according to a mathematical function.

The experiments were performed according to ISO 4049:2009.

### Determination of optimal dimming function and monitoring of instantaneous polymerization shrinkage stress

The optimal mathematical dimming function in the time domain to minimize the shrinkage stress was formulated based on the dependence of shrinkage stress (*f*_*c*_) and the time (*t*) in a photoactivation process by applying constant luminous intensity (*i*). Therefore, the existence of a mathematical function described as *f*_*c*_ (*i,t*) was assumed, which models the shrinkage stress behavior in a conventional photoactivation process. Thus, the shrinkage stress minimization can then be modeled mathematically by an optimization problem of an objective function.

Recent studies have shown that stress data can be displayed as a continuous time function usually for 30-60 min. Measurements for longer periods, such as 24 h, are also feasible due to the stable instrument electronics^6^. However, the major changes in the shrinkage-stress rate occur in the first 5 min (300 seconds). In this way, the behavior of the function *f*_*c*_ for a conventional photoactivation method with constant luminous irradiation (1000 mW/cm^2^ during 35 seconds) was determined by monitoring the shrinkage stress during 300 seconds, of which only the initial 35 seconds (corresponding to the photoactivation interval) were considered for the determination of the minimization function.

The monitoring was performed with a universal testing machine (Emic DL 3000, EMIC Equipamentos e Sistemas de Ensaios Ltda; São José dos Pinhais, Paraná, Brazil). An adaptation using two steel bases was coupled in the arms of the machine, which were adjusted so that the resin could be inserted and polymerized for tests^10^. The gap between the two stents in which the composite was inserted was regulated with a width of 1 mm. Thus, the specimen to be polymerized had the following dimensions: 6 mm × 2 mm × 1 mm. The LED device tip was positioned on the 6 mm side of the steel base, allowing the transmission of light to the full extent of the specimen. The distance of photopolymerization was 2 mm. The device driver software traced the shrinkage stress curve as a function of polymerization time. This experiment was performed in triplicate for each resin brand/model.

Curve fitting by the least squares method was applied to determine the average function , which was considered the objective function for the optimization. The mathematical minimization of the function consisted of calculating the polymerization shrinkage-stress rate (*MPa/s*), obtained by the data differentiation , and with this information, determining the inverse function of the contraction rate, ( )^-1^ . This inverse function was taken as the optimal dimming function to minimize the effects of the polymerization shrinkage. This optimal function was named as the exponential photoactivation method.

The referred optimal function was incorporated in the photopolymerization device, and new experiments for monitoring the shrinkage stress were performed. An average function of the shrinkage stress and its standard deviation was determined for each brand/model of resin. The results from these new experiments showed the patterns of the composite shrinkage when the exponential photoactivation method (*i*_*opt*_) was applied. These results were then compared with the conventional photoactivation method by applying the T-test for equal sample sizes and equal variance. The level of significance for these statistical tests was 0.05.

### Evaluation of cured resin quality

Analyses to determine the conversion degree of the monomers into polymers were applied to evaluate the quality of the cured composites. A stainless-steel matrix with a resin insertion cavity was used to perform this experiment. The composite resins were packed on the cavity in a single increment. They were then photoactivated using the conventional and exponential methods. Five specimens were made for each investigated group. The produced samples dimensions were the same as in the shrinkage stress tests (6 mm × 2 mm × 1 mm). After the photoactivation, the specimens were removed from the mold and stored in a dry mean, in dark containers, at 37°C (±1°C) for 24 h. The specimens were then pulverized into a fine powder. The pulverized composite resins were maintained in a dark box until the Fourier-transform infrared spectroscopy (FT-IR) analysis. Five milligrams of the ground powder was thoroughly mixed with 100 mg of the KBr powder salt^1^ ^,^ ^8^ ^,^ ^14^. The degree of conversion (DC) measurements were performed with a spectrophotometer (Nicolet Nexus 470, Thermo Fisher Scientific; Vernon Hills, Illinois, USA) and were recorded in the absorbance, operating under the following conditions: 50 scans, a 4 cm^–1^ resolution and a 300 to 4000 cm^–1^ wavelength. The DC was determined according to equation:

$$DC=\left(1-\frac{{\left(I_{1638{\mathrm{cm}}^{-1}}/I_{1608{\mathrm{cm}}^{-1}}\right)}_{cured}}{{\left(I_{1638{\mathrm{cm}}^{-1}}/I_{1608{\mathrm{cm}}^{-1}}\right)}_{uncured}}\right)\cdot 100$$ (1)

where the percentage of unreacted carbon-carbon double bonds (%C=C) was determined from the ratio of the absorbance intensities of aliphatic C=C (*I*_1638cm_-1) and aromatic C–C (*I*_1608cm_-1), before and after curing of the specimen^13^. The obtained DC results for each photoactivation technique were compared by applying the T-test for equal sample sizes and equal variance. The level of significance for these statistical tests was 0.05.

### Evaluation of adhesion interface between tooth and resin

Healthy molar human teeth (extracted by orthodontics indication) were used to evaluate the adhesion interface between tooth and polymerized composites. All patients who provided teeth for the present study completed a written consent form. Two teeth were used for each composite brand/model and photoactivation method, resulting in 20 samples for this analysis. Cavities with dimensions similar to the specimens in the previous experiments (6 mm × 2 mm × 1 mm) were made in each tooth. The cavities preparation was performed using water-cooled diamond burs (4138, KG Sorensen; Barueri, São Paulo, Brazil), as it is conventionally performed in dental restorations. A treatment with phosphoric acid was performed under each cavity and the remaining structure was conditioned by a primer adhesive solution (Adapter Single Bond 2, 3M ESPE; Sumaré, São Paulo, Brazil). The resins were packed in the teeth cavity in a single increment and then photoactivated for 35 s using the conventional or the exponential method. The samples were later stored in 2% chlorhexidine solution for 1 week. Glutaraldehyde and sodium hypochlorite were used as disinfectants after tooth extraction.

For the SEM analyses, a longitudinal cut was made in each restored tooth using a diamond cutting disc (Arotec; Cotia, São Paulo, Brazil) at 100 rpm. Note that, after the cutting, the specimens did not have their surfaces polished because the material removed from the teeth in this process could cover the possible cracks in the interface of restoration.

Afterwards, sputter coating for SEM was performed by applying an ultra-thin coating of electrically-conducting metal (gold, in this case) under each specimen. This process was performed to make it susceptible to the emission of secondary electrons to obtain images by SEM (LS-15 EVO, Zeiss; Jena, Thuringia, Germany).

## Results

**Figure 1:**
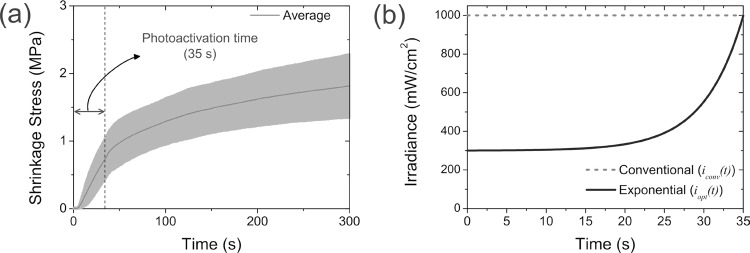
Illustration of: (a) shrinkage stress at the time domain for the conventional photoactivation method; (b) the conventional method of photoactivation and theoretical optimized curve to minimize the effects of the polymerization shrinkage

The monitoring of shrinkage stress showed the function of the conventional polymerization process (i.e., photoactivation with a constant luminous intensity of 1000 mW/cm^2^ for 35 s of irradiation) presented a logarithmic behavior, as illustrated in Figure 1 (a). The graph showed that the resins were under an intensive stress in the initial seconds of the polymerization process, followed by stabilization, where the stress tends to become constant. This behavior shows that the composite contraction occurs at the beginning of the polymerization process.

The experimental data from the shrinkage stress can be fitted by a first order exponential decay function:

$$\bar{f_{c}}\left(1000,\;t\right)=-8.45\cdot e^{\left(\frac{-t}{49.27}\right)}+7.5,$$ (2)

in which the determination coefficient is *R* ^2^=0,99.

The mathematical optimization resulted in a minimization function with exponential behavior:

$$i_{opt}\left(t\right)=a\cdot e^{\frac{t}{b}}+i_{0}$$ (3)

where the parameters *a*, *b* and *i*_0_ can be adjusted to attempt the manufacturers’ recommendations for the photoactivation process. In this work, these parameters were adjusted as follows: *a=0.58309*, *b=4.928* and *i*_0_=300. This function represents an exponential growth of the luminous intensity in the photoactivation time domain. Figure 1 (b) shows this exponential curve, which represents the theoretical optimal curve to minimize the effects of polymerization shrinkage. In this figure, the curve for the conventional method of photoactivation was inserted for comparison purposes.

**Figure 2:**
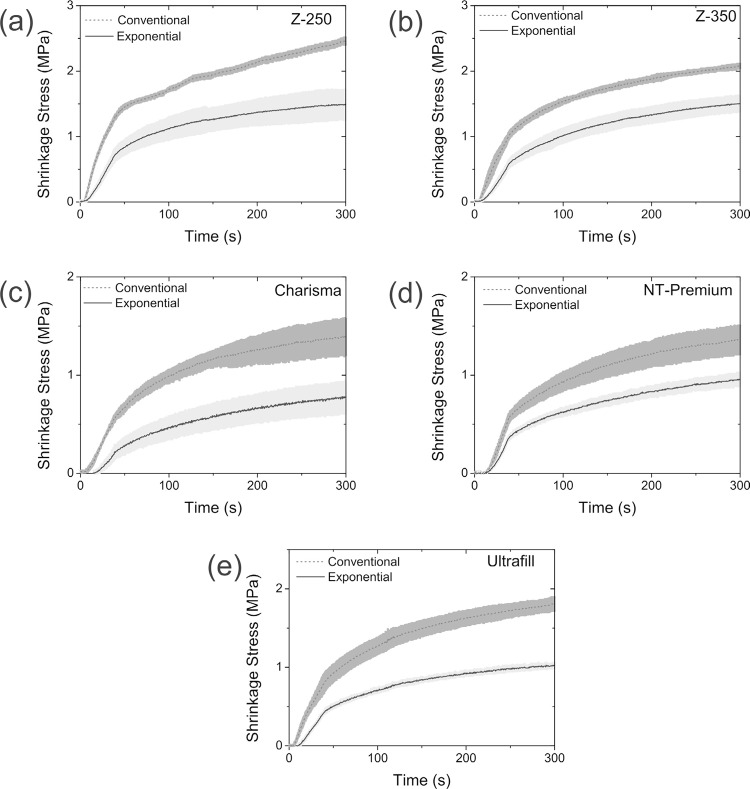
Comparison of the shrinkage stress between the conventional and exponential methods for composite resins: (a) Z-250; (b) Z-350; (c) Charisma; (d) NT Premium and (e) Ultrafill

By applying the exponential photoactivation method, new contraction stress patterns for the resins were obtained. Figure 2 shows comparative graphs of the shrinkage stress for the conventional and exponential photoactivation methods. By analyzing the standard deviation of the shrinkage stress quantification for the conventional and exponential photoactivation methods at the end of the monitoring experiment (300 s) and the T-test result for these values (Table 1), it can be concluded that the exponential method promoted a significant reduction of the shrinkage stress (~36.88% ±6.56) for all analyzed composites brand/models.

**Table 1:** Comparison of the shrinkage stress and degree of conversion for the conventional and exponential photoactivation methods

Composite	Maximum Shrinkage Stress (MPa)		Degree of Conversion (%)	
	Conventional	Exponential	Conventional	Exponential
Z-250	2.46±0.06	1.48±0.23	59.72±4.97	64.46±7.01
Z-350	2.08±0.03	1.50±0.12	62.03±3.23	58.87±4.45
Charisma	1.39±0.18	0.78±0.15	63.86±2.99	64.64±2.65
NT Premium	1.36±0.15	0.96±0.07	79.22±5.02	66.39±6.92
Ultrafill	1.81±0.09	1.02±0.03	67.58±5.44	65.54±3.37
T-test	True difference in mean is not equal to 0		No significant differences	
	(t=6.87, p-value=0.0023)		(t=0.86, p-value=0.44)	

The result from T-test applied on DC values showed there are no significant differences comparing the conventional and exponential photoactivation methods. These results are also synthesized in Table 1.

**Figure 3:**
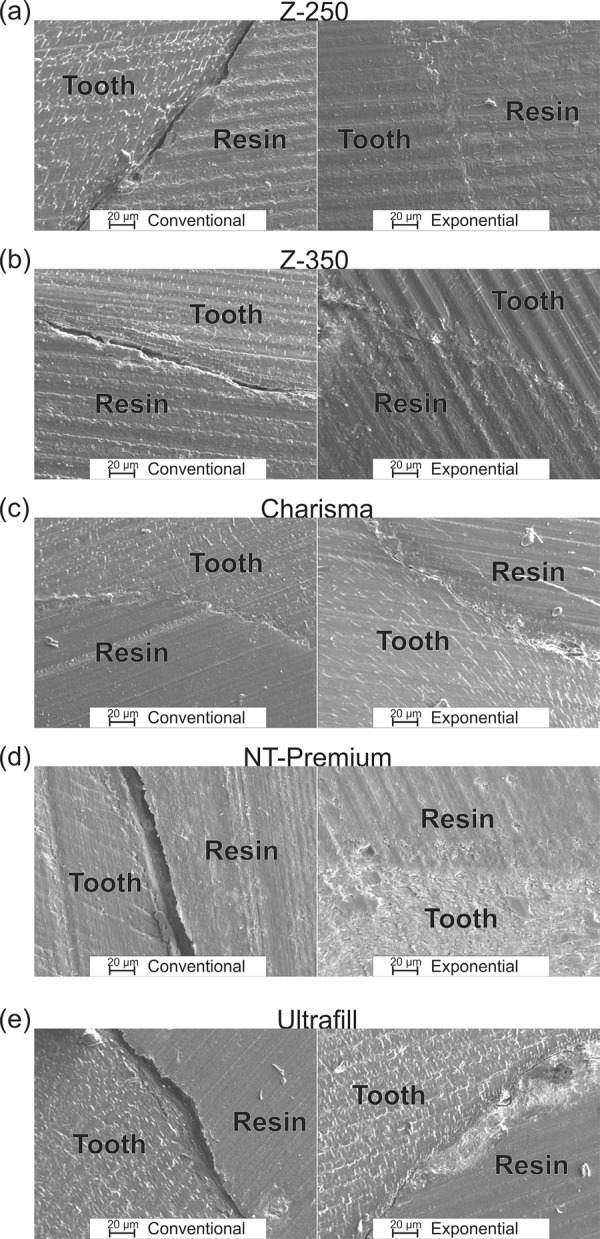
Scanning electron microscopy analyses of the conventional and exponential photoactivation methods for composite resins: (a) Z-250; (b) Z-350; (c) Charisma; (d) NT Premium and (e) Ultrafill

Figure 3 shows the SEM analyses for all resins in this study, comparing the two photoactivation methods. The SEM analyses showed that the reduction of the shrinkage stress by the exponential method promoted the reduction of cracks at the resin/tooth interface for the Charisma and Ultrafill resins. In the other cases, i.e., for the Z250, Z350 and NT Premium composites, the stress reduction was able to eliminate adhesion failures. In the supplementary files, a SEM with lower magnification is shown, where it is observed that the elimination of cracks occurs throughout the extension of the adhesion interface between the tooth and the resin.

## Discussion

The results from the monitoring shrinkage stress showed that the exponential method significantly reduces the stress for all resin models tested in this work. This result can be interpreted because of the control of luminous intensity during the photoactivation that can change the polymerization reaction kinetics. The reduced irradiance at the beginning of the photoactivation process can extend the pre-gel phase, where the material may flow and undergo molecular rearrangement, compensating shrinkages forces^19^.

Similar results were obtained from the soft-start photoactivation protocol, which consists of an initial light exposure with reduced irradiance for a certain time period, followed by full irradiance. However, the soft-start technique does not have a rigid mathematical standardization for the contraction stress function optimization and, because of this, the results obtained from this technique are not as significant as those presented in this study. On this basis, the exponential photoactivation method can improve the marginal adaptation of composite restorations^16^.

The reduction of the contraction stress by itself does not characterize an improvement in the polymerization process of the composite resins. In some situations, the low contraction stress may be associated with partial polymerization of the composite. In this context, the DC results showed there are no statistically significant differences for the ratio between residual monomers for conventional and exponential photoactivation techniques. Although this result is, by itself, very important, we should consider that the DC might be associated with the hardness of the cured resin composite, since there is a high correlation between the two methods^7^ ^,^ ^18^. From this, it can be inferred that mechanical properties of the composite resins photoactivated by the proposed method are similar to those of the resins photoactivated by the conventional method. The DC results also show that the irradiance used in the exponential light curing procedure provides sufficient energy for the adequate resins polymerization.

Finally, the SEM images show that exponential photoactivation technique reduces or even eliminates adhesion failures between the photopolymerized resins and the teeth. We can note a significantly different behavior of the composites photoactivated for the two presented methods and for all the composite resins in this study. Considering the NT Premium and Ultrafill composites, the dimension of the gap at the resin/tooth interfaces for the conventional photoactivation method was large enough (~10 µm) for microleakage and even accumulation of bacteria, including *Streptococcus mutans*. However, concerning the exponential method, only the Charisma and Ultrafill composites showed adhesion failures. Meanwhile, the size of these gaps is lesser than that of the caries-causing bacteria. This result has significant relevance and shows that the technique is feasible for practical application.

In addition, note that we only have studied the effects of a function for irradiance modulation in a fixed activation time (35 seconds). Therefore, the conventional and exponential methods of photoactivation provide different total radiant exposure values, being 35 J/cm^2^ for the conventional method and ~14 J/cm^2^ for the exponential method. This implies that these two methods cannot be analyzed in terms of the Law of Reciprocity^12^ ^,^ ^20^, which we have considered as limitation of our study. Future works can be performed by varying the activation time for the exponential and/or conventional methods, so that the photoactivation energy could be the same. Another proposal for future studies is to compare the exponential method with soft-start techniques, which, as cited in the introduction, have been applied to minimize the polymerization shrinkage.

We still have considered as a limitation of our study the fact that the polymerization shrinkage may be associated with other not analyzed parameters, for example, intrinsic factors such as viscosity, monomers and fillers, and extrinsic factors such as temperature and irradiation time^12^. Thus, future works may use the exponential photoactivation to compare the efficacy of the method from the point of view of these cited parameters and analyzing other mechanical properties as flexural resistance and depth of cure.

## Conclusions

The results obtained in this study showed that the control of irradiance during the photoactivation process by an optimized mathematical function is more efficient than the conventional technique for reducing the effects of polymerization shrinkage in composite resins. According to the DC results, the proposed photoactivation method does not cause damage to the cured resin, which is effective in converting monomers into polymers at the same level as for the conventional method. By the SEM analyses, we concluded that the exponential photoactivation method can reduce or eliminate the adhesion failure between the composite and the tooth.
